# Comparative evaluation of open source software for mapping between metabolite identifiers in metabolic network reconstructions: application to Recon 2

**DOI:** 10.1186/1758-2946-6-2

**Published:** 2014-01-27

**Authors:** Hulda S Haraldsdóttir, Ines Thiele, Ronan MT Fleming

**Affiliations:** 1 Center for Systems Biology, University of Iceland, Sturlugata 8, IS-101 Reykjavik, Iceland; 2 Luxembourg Centre for Systems Biomedicine, University of Luxembourg, 7, avenue des Hauts-Fourneaux, L-4362 Esch-sur-Alzette, Luxembourg

**Keywords:** Metabolic network reconstruction, Metabolite identifiers, Automation, MetMask, The Chemical Translation System, UniChem, Recon 2

## Abstract

**Background:**

An important step in the reconstruction of a metabolic network is annotation of metabolites. Metabolites are generally annotated with various database or structure based identifiers. Metabolite annotations in metabolic reconstructions may be incorrect or incomplete and thus need to be updated prior to their use. Genome-scale metabolic reconstructions generally include hundreds of metabolites. Manually updating annotations is therefore highly laborious. This prompted us to look for open-source software applications that could facilitate automatic updating of annotations by mapping between available metabolite identifiers. We identified three applications developed for the metabolomics and chemical informatics communities as potential solutions. The applications were MetMask, the Chemical Translation System, and UniChem. The first implements a “metabolite masking” strategy for mapping between identifiers whereas the latter two implement different versions of an InChI based strategy. Here we evaluated the suitability of these applications for the task of mapping between metabolite identifiers in genome-scale metabolic reconstructions. We applied the best suited application to updating identifiers in Recon 2, the latest reconstruction of human metabolism.

**Results:**

All three applications enabled partially automatic updating of metabolite identifiers, but significant manual effort was still required to fully update identifiers. We were able to reduce this manual effort by searching for new identifiers using multiple types of information about metabolites. When multiple types of information were combined, the Chemical Translation System enabled us to update over 3,500 metabolite identifiers in Recon 2. All but approximately 200 identifiers were updated automatically.

**Conclusions:**

We found that an InChI based application such as the Chemical Translation System was better suited to the task of mapping between metabolite identifiers in genome-scale metabolic reconstructions. We identified several features, however, that could be added to such an application in order to tailor it to this task.

## Background

Metabolic network reconstructions are knowledge bases of key components in the metabolic reaction network of a particular organism or cell type [1,2]. Reconstructions vary in size from small-scale reconstructions where only a few pathways of interest are included, to genome-scale reconstructions where the aim is to include all known components in a network. The components of a metabolic network reconstruction are the metabolic reactions in the network, the metabolites that participate in those reactions, the enzymes that catalyze the reactions, and the genes that encode those enzymes. Individual components are linked with mathematical structures that enable computational analysis of the network as an integrated system. Computational analysis of genome-scale metabolic reconstructions has, for example, been used for biochemical [3,4], biomedical [5-7], and bioengineering [8,9] purposes.

An important step in the reconstruction of a metabolic network is the annotation of reconstruction components [2]. The identities and functions of components included in metabolic network reconstructions are generally known. Annotation here refers to the process of attaching various metadata for components to a reconstruction. These annotations serve to unambiguously identify components and enable efficient mapping of data to the reconstruction for analysis. Components are generally annotated with an appropriate type of identifier, e.g., an Entrez gene ID for genes and an Enzyme Classification (EC) number for enzymes. Metabolites are usually annotated with several types of identifiers. A comprehensive protocol for genome-scale metabolic reconstruction [2] recommended annotating metabolites with a primary identifier in at least one of the following three databases: ChEBI [10], KEGG Compound [11,12], or PubChem Compound [13]. The protocol also recommended annotating metabolites with structure-based identifiers, such as the IUPAC International Chemical Identifier (InChI) and the Simplified Molecular-Input Line-Entry System (SMILES). We add that further annotation with primary identifiers in databases that are specific to the reconstructed organism is also advisable, e.g., the Human Metabolome Database (HMDB) [14,15] for human metabolic reconstructions.

Database identifiers have the advantage of providing direct links to data that are stored in each database. Data types that can be mapped to the reconstruction via metabolite identifiers include physicochemical data, metabolomics data, metabolic pathways and metabolite structures. Different types of data are available in each database. Whereas KEGG has more data on metabolic genes, enzymes and reactions, HMDB provides more information on metabolites. Since not all chemical databases provide cross-references to all other databases, it is usually not enough to annotate metabolites with only one type of identifier. Instead they should be annotated with identifiers in as many relevant databases as possible. Multiple annotations also aid in identification since any one database may not contain all metabolites in a given reconstruction.

Advantages of structure-based identifiers are that they are unambiguous and database independent. InChIs and SMILES strings can also be converted to metabolite structures that can be used directly for various computational analyses [16-19]. Although SMILES strings have a simpler syntax and are more human readable, InChIs are preferable in many ways [20]. Firstly, they have a layered structure that makes them highly flexible and easy to manipulate. Secondly, they can account for tautomerism. Multiple tautomers of the same compound can be represented with the same standardized or “standard” InChI. Alternatively, a specific tautomer can be represented with a nonstandard InChI. A third advantage of InChIs is that the InChI algorithm in non-propriatory and is implemented in open source software. Version 1 of the InChI is currently in use.

A disadvantage of the InChI is that its length increases with molecular size and level of structural detail. Also, it includes non-alphabetical characters such as /, \, - and +. These features make the InChI inconvenient for internet and database searches [20]. A hashed version, the InChIKey, was therefore created [20]. The InChIKey has a fixed length of 27 characters, and only includes uppercase English letters and dashes. These features also make it a good choice as a database independent identifier for metabolites in metabolic reconstructions. Most chemical databases now include an InChIKey (as well as an InChI and SMILES string) in each database entry. The hashing algorithm that generates InChIKeys from InChIs is not reversible [20], meaning that there is no algorithm that can convert an InChIKey back to an InChI. InChIKeys are therefore not directly convertible to metabolite structure. To retrieve an InChI from an InChIKey it is necessary to use a lookup table or a chemical structure resolver such as the Chemical Identifier Resolver [21] or ChemSpider [22].

Genome-scale reconstructions usually include hundreds of metabolites. The latest human reconstruction, Recon 2, includes over 2500 metabolites [23]. Manual annotation of such a large number of metabolites, with multiple identifiers each, is extremely laborious. Metabolites in early reconstructions were generally annotated manually. Today, reconstruction tools such as the Model SEED [24], rBioNet [25] and the SuBliMinaL Toolbox [26] facilitate the process by populating new reconstructions with pre-annotated components from source databases. Metabolites in the source databases, however, may have incomplete or incorrect annotations, which will then be propagated to all new reconstructions. Metabolites in existing reconstructions may likewise have incomplete or incorrect annotations. Metabolite annotations in metabolic reconstructions may therefore need to be updated prior to their use. Software applications that enable automatic updating of annotations are desirable.

The SuBliMinaL Toolbox comes with an annotation module that can be used to retrieve ChEBI identifiers for metabolites by searching the ChEBI database with metabolite names. A recently published annotation tool called Metingear [27] also features a name search for database identifiers, but against multiple databases. In our experience, metabolite names are poor identifiers. Most metabolites have several synonyms and databases differ in the use of those synonyms. Searching a database with a metabolite name may therefore yield no results if the metabolite is registered under a different synonym. The same name is also often associated with multiple stereoisomers of the same compound, across different databases and within the same database. The name dextrose, for example, is associated with four entries in PubChem Compound: D-glucose (PubChem Compound ID (CID) 5793), *α*-D-glucose (PubChem CID 79025), *β*-D-glucose (PubChem CID 64689) and a generic hexopyranose (PubChem CID 206). As the last entry (PubChem CID 206) demonstrates, names are also sometimes associated with the incorrect structures. Numerous other examples of incorrect associations between names and structures are given in [28]. A name search can therefore yield a list of several candidate identifiers that must be sorted through manually to find the one that best matches the target compound. It is therefore not conducive to automatic updating of identifiers.

A name search is the best option available for updating identifiers for metabolites that are only annotated with a name. However, most metabolites in source databases for metabolic reconstruction tools are annotated with at least one identifier besides name. The same goes for metabolites in existing metabolic reconstructions, especially those that were built according to the aforementioned protocol [2]. The non-name identifiers are generally more specific than names as they refer to specific structures or database entries. Software applications that enable mapping between non-name identifiers could therefore facilitate automatic updating of metabolite identifiers in metabolic network reconstructions.

The problem of annotating large sets of metabolites is well known in metabolomics and chemical informatics. Applications that can be used to partially automate annotation have been developed for these fields. We searched among these for applications that were suitable for mapping between metabolite identifiers in metabolic reconstructions. We only considered open-source applications as these can readily be adapted to the needs of the metabolic reconstruction community and integrated into metabolic reconstruction tools. Three applications that met these criteria were MetMask [29], the Chemical Translation System (CTS) [30] and UniChem [31]. These applications implement annotation strategies that go beyond name search. They enable mapping between multiple types of identifiers, including chemical names. Different annotation strategies are implemented in each of the three applications. Here, we compare these applications, to determine which annotation strategy is best suited for annotation of metabolites in genome scale metabolic reconstructions. We then apply the top application to update annotations of metabolites in the latest human reconstruction Recon 2 [23].

### Applications

#### MetMask

MetMask [29] is a desktop application for creating and querying custom local databases of identifier groups or “metabolite masks”. Identifier groups from multiple sources, such as public databases and private chemical libraries, can be imported into the same MetMask database. We imported identifier groups from Recon 2, HMDB and ChEBI. MetMask merges groups that are deemed compatible by the applications heuristics. A MetMask database can be queried with an identifier of one type (e.g., synonym) to find other identifiers of either the same or a different type (e.g., InChIKey) that belong to the same mask. Metmask is available from http://metmask.sourceforge.net/.

#### The Chemical Translation System

CTS [30] is a web application for mapping between chemical identifiers. It covers 215 types of identifiers, including chemical names, structure-based identifiers, and database identifiers. Queries are sent to a single database where data from multiple external databases has been aggregated. Identifiers are matched based on “standard” InChIKeys, which are generated from “standard” InChIs, i.e., InChIs produced with standard options settings. Standard InChIs and InChIKeys are not tautomer specific. CTS finds all standard InChIKeys that are linked to an input identifier and returns all identifiers of the requested output type(s) that are linked to the same standard InChIKeys. Web services and a web user interface for CTS are available at http://cts.fiehnlab.ucdavis.edu.

#### UniChem

UniChem [31] is a web application that was designed for automatic generation of cross-references between different databases, but can also be used to map between chemical identifiers. It is similar to CTS in that identifiers are matched based on standard InChIs and InChIKeys. Queries to UniChem are also sent to a single database where data from multiple external databases has been aggregated.

There are two major differences between UniChem and CTS that are relevant to metabolite annotation. The first is that UniChem covers a much lower number of identifier types. At the time of writing, it covered identifiers in 18 public databases in addition to standard InChIs and InChIKeys. Covered databases included KEGG Compound, ChEBI and HMDB, but not PubChem Compound. Chemical names were not covered. The second major difference between UniChem and CTS is that data from external databases must pass various quality checks before they are imported into the UniChem database. External database entries are generally required to include at least a database identifier and a standard InChI to be included in the UniChem database. The quality checks performed by UniChem include checking whether a standard InChI in an entry can be converted to a standard InChIKey. If the entry also includes a standard InChIKey, it is checked against the standard InChIKey generated from the standard InChI. Entries that fail these checks are excluded from the UniChem database as either the standard InChI, standard InChIKey, or both can then be assumed to be invalid. In addition to these quality checks, UniChem keeps track of which database identifiers are currently associated with a given InChI and which identifiers were associated with that InChI in the past. It does not output obsolete associations unless it is requested by the user.

These differences between UniChem and CTS stem from the fact that they were designed for different purposes. CTS was designed for metabolite annotation and emphasizes coverage, whereas UniChem was designed for automatic database cross-referencing and emphasizes specificity. We include UniChem here to assess the value of UniChem-like quality checks in metabolite annotation. Web services and a web user interface for UniChem are available at https://www.ebi.ac.uk/unichem/.

## Results

### Identifier mapping tests

**Figure 1:**
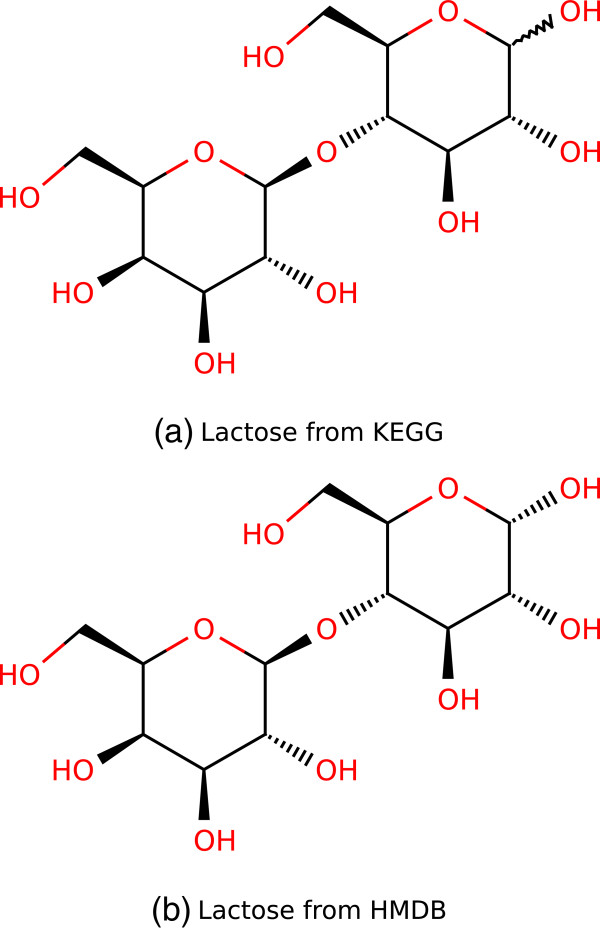
**Lactose stereoisomers.** Two epimers of lactose occur in nature, *α*-lactose and *β*-lactose. The epimers differ by the configuration of structural groups around a single stereogenic carbon atom (top right). **(a)** In KEGG Compound the synonyms lactose and milk sugar are assigned to a generic stereoisomer, where the configuration around this stereogenic carbon is not specified (C00243). Reactions, enzymes and pathways involving lactose are linked to this entry in KEGG. **(b)** The same synonyms and most lactose-related data are linked to the *α*-epimer in HMDB (HMDB00186). There is neither an entry for the generic stereoisomer in HMDB, nor an entry for the *α*-epimer in KEGG Compound.

The tests described in *Methods* revealed a number of differences between the three mapping applications. The overall performance of each application is quantified in Table 1. Performance on individual mapping tests is given in Additional file 1: Table S1. UniChem performed best on tests involving identifier types that it covered. UniChem generally only returned the preferred output identifier, for each input identifier that was associated with at least one output identifier in the UniChem database. Many input identifiers, however, were not associated with any output identifier. This was also the case for CTS, which indicates that it is a characteristic of InChI based mapping strategies. The fact that no output identifier of a particular type is returned for a given input identifier does not necessarily mean that the input identifier is not in the database. It only means that no identifier of the requested output type is associated with the exact same standard InChI. A single compound can have more than one valid structure, each represented with a distinct standard InChI. The distinct, equally valid structures are usually stereoisomers. If two databases associate different stereoisomers with their respective entries for the same compound, the two entries cannot be linked through InChIs. The KEGG Compound and HMDB entries for lactose are good examples of this (see Figure 1). Because the lactose stereoisomers in the two entries have different InChIs, neither UniChem nor CTS can map between them.

**Table 1:** Quantified overall performance of the three mapping applications

	**Mean counts**				
	**In**	**Hits**	**Out**	**Matches**	**Mean score**
MetMask	99	98	146	93	0.63
CTS	99	80	105	75	0.57
UniChem	99	74	77	72	0.70

Scores were calculated as described in Section *Scoring*. Means were taken over all input-output pairs that were covered by each application. The means reflect well general characteristics of each application observed in individual tests (see Additional file 1: Table S1).

**Figure 2:**
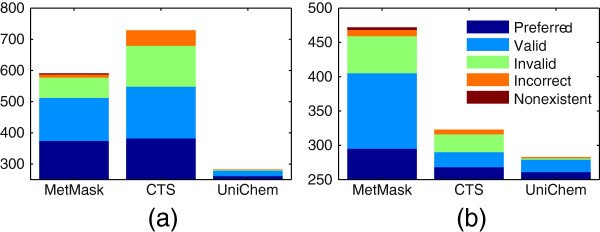
**Identifiers output in identifier mapping tests.** Annotations of unique identifiers returned by each application, **(a)** when all mapping tests are included, and **(b)** when only tests involving identifier types covered by UniChem are included. The output identifiers returned in all included tests were pooled and duplicates removed. If the same identifier was returned in more than one test it was only counted once. The annotations are explained in Section *Scoring*.

On average, a slightly higher number of input identifiers were associated with at least one output identifier with CTS than with UniChem. CTS, however, also returned a higher number of non-preferred identifiers, and thus received a lower overall score (Table 1). These differences between CTS and UniChem are attributable to two factors; the greater number of identifier types covered by CTS, and the checks implemented by UniChem to prevent errors in their database (see Section *Applications*). The fact that CTS covers chemical names as an identifier type has a particularly large effect. Chemical names are ambiguous identifiers and the same name can be associated with a number of different structures [28]. Using names as input identifiers will therefore often result in a long list of candidate output identifiers (see Additional file 1: Table S1). Referring to the previous example, the name lactose is associated with different structures in KEGG Compound and HMDB. Inputting the name lactose into CTS will return all identifiers that are associated with either structure. The number of incorrect output identifiers returned for names is also generally higher than for other input types, because names are more frequently associated with an incorrect structure [28]. When chemical name is included as an input identifier type (Figure 2a), the number of incorrect identifiers returned by CTS is much greater. When only identifiers that are covered by UniChem are considered (Figure 2b), CTS results are more similar to those of UniChem. The remaining difference between the two applications is presumably due to the quality checks implemented by UniChem.

MetMask returned the greatest number of preferred identifiers on average, but it also returned the greatest number of non-preferred identifiers. It received a score in between those of UniChem and CTS (Table 1). MetMask returned the greatest number of output identifiers on individual mapping tests but when all tests were combined, MetMask returned fewer unique identifiers than CTS (Figure 2). The reason for this is that MetMask generally returns the same set of output identifiers, when it is queried with different input identifiers for the same compound. MetMask will, for example, return the same set of ChEBI ID whether it is queried with the KEGG Compound ID (CID) for lactose or the HMDB ID. The ChEBI ID for both lactose stereoisomers in Figure 1 will be returned for either query. This is a consequence of the way metabolite masks are defined. All identifiers for lactose belong to the same mask. Querying MetMask with any identifier in a particular mask will always yield all identifiers of the requested output type that are in the same mask. Queries with different types of identifiers for the same compounds are therefore not independent. This also explains why the difference between the total number of unique identifiers returned by MetMask, when all tests are included (Figure 2a) and when only UniChem-compatiple tests are included (Figure 2b), is so much smaller than for CTS.

Taken together, these results demonstrate that regardless of the choice of mapping application, some manual effort is required for proper annotation of metabolites with database identifiers. With MetMask this effort is mainly directed at sorting through candidate output identifiers to locate the preferred ones. With UniChem it is directed at searching for missing identifiers. Some effort is required for both sorting and gap filling with CTS, although less for each than with the other two applications. In the next section we seek ways to minimize this manual effort.

### Optimization of performance

In each individual mapping test described in the previous section, a single identifier was used as input for each compound. Metabolites in metabolic reconstructions, however, are often annotated with more than one identifier, e.g., a name and an HMDB ID. Using all available identifiers to map to missing ones should be more powerful than using only one at a time. If one identifier for a given compound does not map to any identifier of the requested output type, a different identifier for the same compound may be used to fill that gap. If multiple identifiers of different types map to the same output identifier, our confidence in that output identifier is increased. Such additive mapping of identifiers is only useful, however, if the different input identifiers do not all yield the same outputs, i.e., if queries with different input identifiers are independent. As discussed in the previous section, this is not the case for MetMask. We therefore only tested this method on CTS and UniChem.

We investigated the effects of combining outputs for two to six different types of input identifiers with CTS and two to four different types of input identifiers with UniChem. The order, in which input identifiers were added, was determined based on the numbers of identifiers already available in Recon 2. All metabolites in Recon 2 have a name, so we initially used only names. In the second iteration, we combined outputs for names and standard InChIKeys, since standard InChIKeys were the second most common identifier type in Recon 2. In each subsequent iteration, we added the next-most-common type of input identifier until outputs for all input types had been combined. After each iteration, we assigned a confidence score to each returned output identifier that was increased each time that same output identifier was returned. For each metabolite, we only retained output identifiers with the highest confidence score out of all identifiers returned for that same metabolite. The confidence score assigned to each output identifier was increased by 0.5, if the identifier was returned with name as the input type and 1 otherwise. Our confidence in identifiers returned for names was lower than for other identifiers because, as discussed above, the number of non-preferred and incorrect identifiers returned for names was higher.

The overall results of each iteration of the additive mapping tests are quantified in Table 2. The mean score for CTS was significantly higher on these tests, than on any individual mapping test (Table 1). In fact, CTS scored higher on the additive mapping tests than any of the three applications did on individual mapping tests, even when outputs for only two types of input identifiers (names and standard InChIKeys) were combined. The mean score for UniChem also increased as input identifiers were added, although less than for CTS. When multiple input identifiers were combined, CTS and UniChem received similar scores, but for different reasons. UniChem continued to return only the preferred output identifier for most metabolites but, even with four input identifier types combined, it did not return an output identifier for all metabolites. CTS, on the other hand, returned at least one candidate output identifier for most metabolites. The preferred identifier was generally amongst those candidates but several non-preferred identifiers were also returned. In our opinion, the results of combining input identifiers were qualitatively better for CTS than for UniChem. Despite the fact that some manual effort is required to sort through candidate output identifiers returned by CTS, it is reassuring to know that the output is relatively comprehensive. Otherwise, it would be necessary to search manually for identifiers that may not even exist. Accepting and rejecting suggested identifiers is fast by comparison. In the following section, we therefore use additive mapping with CTS to update metabolite annotations in Recon 2 [23].

**Table 2:** Quantified overall performance of CTS and UniChem on the additive identifier mapping tests

		**Mean counts**				
		**In**	**Hits**	**Out**	**Matches**	**Mean**
						**score**
Name only	CTS	100	67	141	63	0.30
	UniChem	NA	NA	NA	NA	NA
+ InChIKey	CTS	100	95	112	87	0.
	UniChem	100	80	83	78	0.75
+ ChEBI	CTS	100	96	118	93	0.75
	UniChem	100	84	88	82	0.78
+ HMDB	CTS	100	96	113	89	0.75
	UniChem	100	85	88	82	0.79
+ KEGG	CTS	100	97	116	93	0.78
	UniChem	100	87	91	85	0.82
+ PubChem	CTS	100	97	117	94	0.78
	UniChem	NA	NA	NA	NA	NA

Results of individual tests are given in Additional file 1: Table S2. NA implies that the input identifier type was not covered by the corresponding application. PubChem refers to the PubChem Compound database and KEGG to the KEGG Compound database.

### Update of Recon 2 metabolite annotations

Recon 2 includes 2,626 unique metabolites. During the reconstruction of Recon 2, 1,690 metabolites were annotated with a standard InChIKey, 1,125 with a ChEBI ID, 1,040 with an HMDB ID, 396 with a KEGG CID, and 150 with a PubChem CID. All metabolites were annotated with a metabolite name. We updated metabolite identifiers in Recon 2 using additive mapping with CTS as described in the previous section. We used CTS both to review existing annotations and to add as many new ones as possible. In addition to updating identifier types that were already present in Recon 2, we added identifiers for the LIPID MAPS Structure Database (LMSD) [32] which were previously missing. To speed up the process of updating identifiers, we took advantage of the extensive metabolite annotations that are available in HMDB [14,15]. The step-by-step process is described in Additional file 1: Section 2. We added a total of 3,049 new identifiers to Recon 2, removed 124 incorrect identifiers, and replaced 569 identifiers. We therefore updated a total of 3,746 identifiers. All except 233 identifiers were updated automatically. These 233 identifiers were selected manually from a list of 2,790 candidates that were returned by CTS. Manually sorting through the list of candidates required approximately ten man-hours. A random sample of 100 automatically updated identifiers were also checked manually. All 100 were found to be correct. The full list of updated metabolite annotations in Recon 2 is included in the Additional file 2.

**Figure 3:**
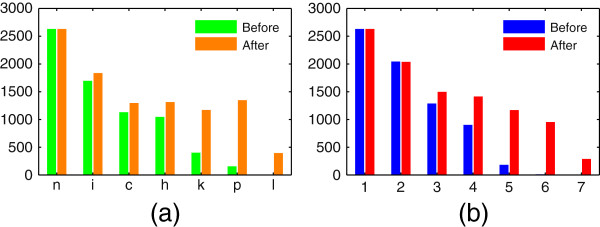
**Recon 2 identifiers.** Identifier statistics for Recon 2 before and after metabolite annotations were updated with CTS. **(a)** Number of unique metabolites with each of the seven types of identifiers. n: names, i: InChIKeys, c: ChEBI ID, h: HMDB ID, k: KEGG CID, p: PubChem CID, l: LipidMAPS ID. **(b)** Number of unique metabolites with one, and up to seven, identifiers each.

The majority (1,962/2,660) of added identifiers were KEGG and PubChem CID (Figure 3a), which were previously lacking in Recon 2. We also added LMSD ID for 389 metabolites. Around half (65/124) of all incorrect identifiers were PubChem CID. The remaining half was evenly distributed among ChEBI ID, HMDB ID and KEGG CID. Incorrect identifiers were identified and removed automatically (see Additional file 1: Section 2). The majority of replaced identifiers (464/569) were ChEBI ID. In most cases, we replaced a ChEBI ID for a charged metabolite, with a ChEBI ID for the same metabolite in its neutral state. ChEBI and PubChem Compound often include separate entries for metabolites in neutral and various charged states, but HMDB, KEGG Compound and LMSD usually only include metabolites in their neutral state. For the sake of mapping and other comparisons between databases, it is therefore preferable to include identifiers for metabolites in their neutral states in metabolic reconstructions. If metabolite charge is required, it can be predicted with software tools, such as ChemAxon’s Calculator Plugins (ChemAxon Kft., Budapest, Hungary).

Most of the added identifiers (2,991/3,049) were for metabolites that were already annotated with at least one identifier besides a chemical name in Recon 2 (Figure 3b). Non-name identifiers were added for 28 metabolites that were previously only annotated with a name. That leaves 594 Recon 2 metabolites with name as the only annotation. There are two possible reasons for this; either these metabolites are not included in the CTS database, or the synonyms used for them in Recon 2 is not included. The CTS name search is currently only capable of matching names exactly, so even slight differences between a metabolite name in Recon 2 and the synonyms listed for that metabolite in CTS would prevent finding a match. A majority (432/594) of the metabolites for which no identifier was found consisted of macromolecules and metabolites with variable structures (i.e., R groups), such as polysaccharides and proteins. Such metabolites are seldom included in the chemical databases considered here, so it is not surprising that no identifiers were found. In addition, metabolites with variable structures cannot be represented with an InChI. An InChI based application such as CTS therefore cannot cover them. The remaining metabolites (162/594) are more likely to be included in the databases considered here. If they are, they must be registered under different synonyms than the names included in Recon 2. Non-name identifiers for these metabolites will need to be searched for manually.

## Discussion

The three applications compared in this work implement two different metabolite annotation strategies. MetMask implements what can be termed a “metabolite masking” strategy (see Section *MetMask*), whereas CTS and UniChem implement two different versions of an InChI based strategy (see Sections *The Chemical Translation System* and *UniChem*). We found the InChI based strategy to be better suited for annotation of metabolites in genome-scale metabolic reconstructions. The main advantage of this strategy over the metabolite masking strategy is that multiple types of information about a metabolite can be used to perform independent searches for missing annotations. The independent search results can then be combined to increase both coverage and specificity. Candidate annotations are thereby found for a greater number of metabolites (increased coverage), while candidate annotations for each metabolite are fewer and usually include the preferred annotations (increased specificity). Candidate annotations, which are returned by multiple independent searches, can additionally be assigned in an automatic manner with more confidence. Neither the CTS nor UniChem user interfaces currently offer the possibility of combining multiple types of information to search for missing annotations. Here we performed each search separately and combined results afterwards. This process was slow and required a considerable amount of programming. An InChI based application that allows simultaneous input of multiple types of metabolite information would greatly simplify and accelerate annotation.

When multiple independent search results were combined, the InChI based strategy implemented in CTS gave qualitatively better results than the version implemented in UniChem. Although UniChem gave more specific results, CTS covered a greater number of metabolites. The main advantage of CTS over UniChem is that it can map between a greater number of identifier types. We chose to map between a limited number of identifier types, that are relevant for the human metabolic reconstruction Recon 2, but the same strategy could be used to map between any of the 215 types of identifiers covered by CTS.

The greater metabolite coverage of CTS was mostly due to the fact that CTS allows chemical names as inputs. This was also the main reason for the lower specificity of CTS results. Chemical names are rather generic metabolite identifiers, or at least they are used rather generically in chemical databases. The same name is often associated with multiple different structures, sometimes incorrectly [28]. When names are input to an InChI based mapping application, identifiers for different but equally valid structures may be returned leading to increased coverage. However, identifiers for nonpreferred, invalid or even incorrect structures may also be returned leading to reduced specificity. Inputting the name lactose to CTS, for example, will return both a KEGG CID and an HMDB ID, for different but equally valid lactose stereoisomers (see Figure 1). However, it will also return a total of four PubChem CID, one of which is invalid as it refers to a generic disaccharide (PubChem CID 294). To retain the coverage obtained with chemical names as inputs, while minimizing the adverse effects it has on specificity, we introduced a confidence score that gave annotations returned for names a lower priority. A similar mechanism could be built into the metabolite annotation application suggested above, where multiple types of metabolite information could be input simultaneously as search criteria.

Although the fact that CTS allows names as inputs explains most of the difference between the specificity of CTS and UniChem, it does not explain all of it (see Figure 2 and Additional file 1: Table S1). Some of this difference is also due to the quality checks performed by UniChem before data from external databases is imported into the UniChem database (see Section *UniChem*). Any InChI based application would benefit from similar quality checks. A recent study [33] showed that different structural representations (Molfiles, InChIs, SMILES) within the same database entry often do not represent the same structure. Such mismatches are indicative of errors in database entries. Quality checks such as the ones implemented in UniChem hinder such errors from being propagated to local databases for annotation applications. Additional quality checks could also be performed, such as checking whether the two dimensional structure (e.g., in Molfile format) and the chemical formula in an external database entry match the standard InChI. Chemical formulas can also be used to check candidate annotations returned for metabolites and to weed out incorrect ones. All metabolites in metabolic network reconstructions should be annotated with their chemical formulas. We used metabolite formulas in Recon 2 to review database identifiers that were added to the reconstruction (see Additional file 1: Section 2). If the metabolite formula in the database entry associated with an identifier did not match the formula in Recon 2, we assumed the identifier was incorrect and rejected it. Differences in numbers of hydrogen atoms between formulas were ignored. The metabolite annotation application suggested above could include metabolite formulas as one type of input information about metabolites. Candidate identifiers associated with different formulas than the input formula would then be rejected before they were added to the reconstruction.

The coverage of any InChI based application is limited to metabolites with defined structures that can be represented with InChIs. Metabolic reconstructions often include generic metabolites with undefined structural elements such as R groups. These generic metabolites represent whole classes of structurally similar metabolites that undergo the same metabolic transformations in vivo. They are introduced into reconstructions for simplification. Such generic metabolites cannot be represented with an InChI and therefore cannot be covered by an InChI based metabolite annotation application. An annotation application based on a metabolite masking strategy would be better suited to mapping between identifiers for such metabolites. The fact that an InChI based application cannot cover generic metabolites of this type does not decrease its value much, since these metabolites are generally a minority of all metabolites in metabolic reconstructions and only a minority of those is expected to be included in chemical databases of interest.

Generic metabolites of a different type that are also found in metabolic reconstructions are generic stereoisomers, i.e., metabolites with undefined stereochemistry at one or more stereocenters. An example found in Recon 2 is lactose in Figure 1a. As enzymes that catalyze metabolic reactions involving lactose generally don’t have known stereospecificity, both the *α*- and *β*-epimers are assumed to participate in the same reactions. Instead of needlessly complicating the reconstruction by writing the same reactions twice, the two epimers are collapsed into a single generic stereoisomer. The metabolic component lactose in Recon 2 therefore encompasses both *α*- and *β*-lactose. Similar examples are frequent in fatty acid metabolism where cis-trans isomerism is not specified unless necessary, i.e., for fatty acids that participate in reactions that are catalyzed by enzymes with known stereospecificity.

Generic stereoisomers can be represented with an InChI and can therefore be covered by an InChI based application. CTS often returned several candidate identifiers for such metabolites that needed to be sorted through manually to select the preferred ones. One reason for this is that the names of generic stereoisomers in Recon 2 are often associated with more specific or even more generic (and thus invalid) stereoisomers in the databases considered here. As discussed above, the name lactose is associated with *α*-lactose (more specific) in HMDB and a disaccharide with no specified stereochemistry (more generic) in PubChem among other database identifiers. Another reason for why CTS often returned several candidate identifiers for generic stereoisomers was that preexisting annotations of these metabolites in Recon 2 were sometimes rather ambiguous. Lactose in Recon 2, for example, was annotated with the KEGG CID for the generic stereoisomer (Figure 1a) and the HMDB ID for the *α*-epimer (Figure 1b). When these two identifiers were used in combination to find the PubChem CID for lactose, CTS naturally returned PubChem CID for both stereoisomers. This raises the question of how generic stereoisomers should be annotated in metabolic reconstructions. The general rule should be to annotate each metabolite with the most generic identifier of each type that is still valid. Lactose therefore should be annotated with identifiers for the generic stereoisomer in Figure 1a. However, there is no entry for this lactose stereoisomer in HMDB. Instead, HMDB includes separate entries for the more specific *α*- and *β*-epimers of lactose. In such cases, the general rule in the past appears to have been to select the identifier for the most prevalent specific stereoisomer, e.g., the HMDB ID for *α*-lactose, as relevant biochemical data is more likely to be associated with that identifier. The advantage of annotating generic stereoisomers with identifiers for more specific ones is precisely that they provide links to such data. The disadvantage is that the identity of metabolic components becomes somewhat ambiguous. It may, for example, not be obvious to all users of Recon 2 whether the metabolic component lactose represents the generic stereoisomer or only *α*-lactose since it is annotated with identifiers for both.

Reconstruction of the metabolic network of an organism or cell type is an iterative process. Recon 2 is the latest iteration of the human metabolic network reconstruction. While Recon 2 is much more comprehensive than its predecessor Recon 1 [34], it probably does not capture the entire human metabolic network and further iterations are expected in the future [23]. Our results suggest some guidelines for researchers to keep in mind when annotating new metabolites that are added to metabolic reconstructions, human or otherwise. Firstly, each metabolite should be annotated with at least one identifier besides name if that is possible. Secondly, each metabolite should generally be annotated with identifiers for its neutral form. Exceptions exist for metabolites that only participate in metabolic reactions in a particular charged state, e.g. inorganic ions such as Cl ^−^ and Mg ^2+^. Thirdly, each metabolite should preferably be annotated with the most generic identifier of each type that is still valid. This also applies to metabolite names. The name D-glucose, for example, should not be used for a metabolic component that is meant to represent *α*-D-glucose or vice versa. If no generic identifier of a particular type is available for a metabolite, a more specific identifier may be used. Researchers should, however, be aware that doing so makes the identity of that metabolite somewhat ambiguous. Best practices would be to include a note that specifies the relationship of an identifier to a metabolic component. The ChEBI ontology could serve as a guideline for how relationships between metabolites should be specified. So, for example, it would be noted that the metabolite with HMDB ID HMDB00186 (Figure 1b) *is a* Lactose.

An InChI based metabolite annotation application has the potential to enable fully automatic mapping between identifiers for metabolites with defined structures. Fully automatic mapping, however, would require that both databases and reconstructions were free of errors and ambiguity. As several authors have demonstrated [28,33,35], errors and ambiguities are quite common in publicly available chemical databases. If a database identifier is associated with an incorrect InChI in a database it will not be mapped to the correct metabolite in a reconstruction. As we demonstrated here, erroneous metabolite annotations are also found in metabolic reconstructions. In particular, we removed 124 incorrect identifiers from Recon 2. As discussed above metabolite annotations in metabolic reconstructions can also be ambiguous, e.g., when a metabolite is annotated with the name of one stereoisomer but the KEGG CID of another. If a metabolite is annotated with an incorrect identifier in a metabolic reconstruction it may not be mapped to the correct database or structure based identifier. Fully automatic mapping between metabolite identifiers will not be possible until such errors and ambiguities are resolved. An InChI based application such as CTS, however, with the modifications suggested above, can significantly reduce the manual effort required for mapping between metabolite identifiers.

## Experimental

CTS was accessed by calling the “Convert” web service as described at http://cts.fiehnlab.ucdavis.edu/moreServices/index. UniChem was accessed by calling the web service method "Get src_compound_ids from src_compound_id" as described at https://www.ebi.ac.uk/unichem/info/webservices. Web services were called from MATLAB (version R2009b, MathWorks, Natick, MA) using the built-in function urlread. MetMask (version 0.5.3) was installed on a desktop computer running Windows 7. MetMask databases were created by importing identifier groups from Recon 2, HMDB and ChEBI. Database queries were formulated as described at http://metmask.sourceforge.net/manual.htmlwith all options set to their default values. Output from all mapping applications was parsed in MATLAB using the built-in function regexp.

## Conclusions

We found that an application implementing an InChI based strategy could facilitate automatic mapping between metabolite identifiers in metabolic network reconstructions. Of the two InChI based applications evaluated here we found CTS to be qualitatively better. The main advantage of CTS is the large number of identifier types it can map between. As CTS is open source it can be adapted to the task of mapping between metabolite identifiers in metabolic network reconstructions with relative ease. We suggest several features that could be added to CTS to optimize its performance on this task. In particular, we suggest combining multiple types of information about metabolites to find new identifiers. A confidence score can be used to account for the fact that some types of input information, in particular metabolite names, are less reliable than others. We further suggest implementing various quality checks, similar to those implemented in UniChem, to limit the number of incorrect identifiers returned for a metabolite. When simple versions of some of the suggested features were implemented, CTS allowed us to update more than 3,500 metabolite identifiers in Recon 2. Most were updated automatically. Based on this experience, we suggest some guidelines for future annotation of metabolites in metabolic network reconstructions. We hope that the updated Recon 2 identifiers will facilitate application of Recon 2 in the future. More generally, we hope that our results will guide developers of reconstruction tools in implementing strategies for automatically updating metabolite identifiers in metabolic network reconstructions.

## Methods

### Design of identifier mapping tests

MetMask and CTS were tested by mapping six types of input identifiers to four types of output identifiers. The six input identifier types were metabolite name, standard InChIKey, ChEBI ID, HMDB ID, KEGG CID and PubChem CID. Output identifier types were the subset of input types that act as primary keys in public databases, i.e., ChEBI ID, HMDB ID, KEGG CID and PubChem CID. Output identifiers of these four types can easily be verified by looking them up in the relevant databases. Since metabolite names and InChIKeys are more difficult to verify, they were not included as output identifier types. In total, we tested MetMask and CTS on 20 pairs of input-output identifier types, since input identifiers were not mapped to output identifiers of the same type. ChEBI ID for example, were only mapped to HMDB ID, KEGG CID and PubChem CID. UniChem was tested on nine input-output pairs, since it does not cover metabolite names or PubChem CID. All three applications were tested on a set of 100 metabolites from the human metabolic reconstruction Recon 2 [23]. The metabolites were chosen randomly from the subset of Recon 2 metabolites that were already annotated with at least two of the four database identifiers. For each metabolite, we verified the existing annotations and attempted to fill in missing annotations of the remaining four input types. The end result was 100 metabolite names, 100 InChIKeys, 98 ChEBI ID, 100 HMDB ID, 97 KEGG CID and 100 PubChem CID. A minimum of five identifiers were located for each of the 100 test metabolites.

### Scoring

**Figure 4:**
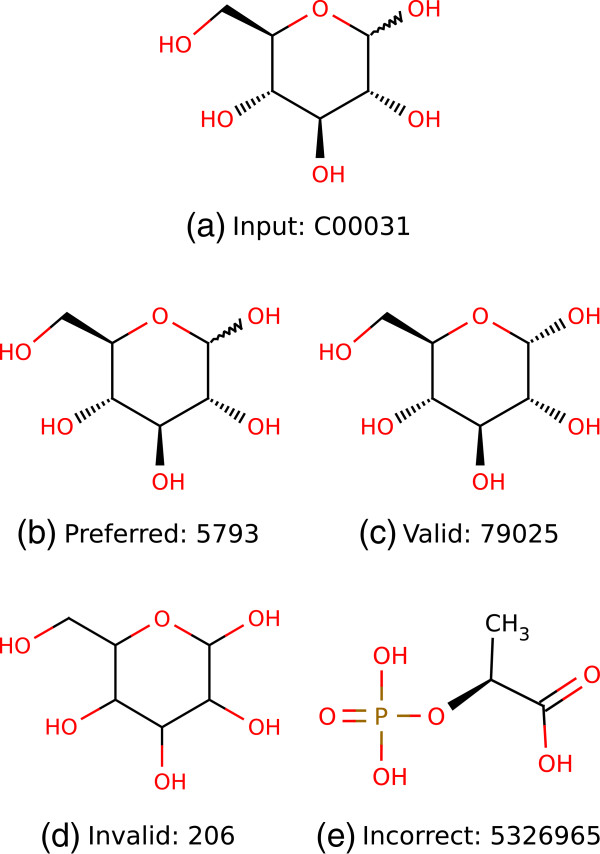
**Annotation of output identifiers.** An example demonstrating annotation of output PubChem Compound identifiers (b-e), when the KEGG Compound identifier for D-glucose **(a)** is input to a mapping application. The preferred output identifier is for D-glucose **(b)**, but an identifier for alpha-D-glucose **(c)** is also valid since it is a D-glucose. An identifier for a generic hexose **(d)**, however, is not valid. Finally, an identifier for phospholactic acid **(e)**, which is a completely different compound, is incorrect.

To quantify the relative performance of the three mapping applications, we devised a simple scoring system. For each input-output pair, each mapping application returned a list of candidate output identifiers associated with the set of input identifiers. The number of output identifiers associated with a single input identifier ranged from zero to several. We annotated each returned output identifier as *preferred*, *valid*, *invalid*, *incorrect* or *nonexistent* (see Figure 4 for an example). Preferred identifiers point to the preferred stereoisomer of each compound, which is generally the same as the input stereoisomer. There is exactly one preferred output identifier for each input identifier. Valid identifiers point to valid but not preferred stereoisomers, invalid identifiers point to invalid stereoisomers or mixtures, incorrect identifiers point to different compounds, and nonexistent identifiers do not point to anything. Once all output identifiers had been annotated in this manner, we calculated a score based on the number of input identifiers (In), the number of input identifiers for which at least one output identifier was returned (Hits), the total number of returned output identifiers (Out), and the number of preferred output identifiers (Matches). The score was calculated as

(1) $$\text{Score}=\frac{\text{Hits}\times \text{Matches}}{\text{In}\times \text{Out}}.$$

This score can range from 0 to 1. An application receives the maximum score if it returns the preferred output identifier, and no other, for each input identifier. It receives a lower score if it returns non-preferred identifiers, or none at all, for a subset of input identifiers, since some manual effort is then required to sort through results and fill gaps. Note that the number of input identifiers (In) varies between identifier types, because two of the 100 test metabolites were not found in ChEBI (In=98) and three were not found in KEGG Compound (In=97).

## Competing interests

The authors declare that they have no competing interests.

## Authors’ contributions

All authors conveived and designed performance tests. HSH performed the tests and analyzed results. HSH updated metabolite annotations in Recon 2. All authors wrote the manuscript. All authors read and approved the final manuscript.

## Supplementary Material

Additional file 1 Supplementary tables and text. Click here for file

Additional file 2 A full list of updated metabolite annotations in Recon 2. Click here for file
