# Supplementary material for: Comparative evaluation of open source software for mapping between metabolite identifiers in metabolic network reconstructions: application to Recon 2
Source: J Cheminform. 2014 Jan 27;6:2. doi: 10.1186/1758-2946-6-2 (PMC3917611; doi:10.1186/1758-2946-6-2)
Supplement: Additional file 1 — Supplementary tables and text. [file 1758-2946-6-2-S1.pdf]

Comparative evaluation of open source software for mapping between metabolite identifiers in metabolic network reconstructions: application to Recon 2

Supplement

Hulda S. Haraldsdóttir[1], Ines Thiele[1,2] and Ronan M. T. Fleming[1,2,†]

1 Center for Systems Biology
University of Iceland, Reykjavik, Iceland

2 Luxembourg Centre for Systems Biomedicine
University of Luxembourg, Esch-sur-Alzette, Luxembourg

† E-mail: ronan.mt.fleming@gmail.com

January 18, 2014

1 Supplementary tables

Table S1: Results of individual identifier mapping tests. Rows are for input identifier types and columns for output types. PubChem refers to the PubChem Compound database and KEGG to the KEGG Compound database. For each input-output pair we counted the number of input identifiers (In), the number of input identifiers for which at least one identifier was output (Hits), the total number of output identifiers (Out), and the number of preferred output identifiers (Matches). Results are given for MetMask/CTS/UniChem. NA implies that the input identifier type, output identifier type, or both were not covered by the corresponding application.

		Output type			
Input type	Count	ChEBI	HMDB	KEGG	PubChem
Name	In	100/100/NA	100/100/NA	100/100/NA	100/100/NA
	Hits	99/66/NA	100/67/NA	98/65/NA	98/70/NA
	Out	233/144/NA	119/78/NA	123/106/NA	116/235/NA
	Matches	98/59/NA	100/65/NA	97/63/NA	79/65/NA
InChIKey	In	100/100/100	100/100/100	100/100/100	100/100/NA
	Hits	99/76/68	100/96/100	98/74/73	98/98/NA
	Out	230/91/76	119/96/100	123/92/74	116/115/NA
	Matches	98/71/63	100/94/98	97/73/72	79/79/NA
ChEBI	In		98/98/98	98/98/98	98/98/NA
	Hits		98/68/65	96/79/69	96/96/NA
	Out		117/68/65	121/100/70	114/114/NA
	Matches		98/68/64	95/79/69	78/88/NA
HMDB	In	100/100/100		100/100/100	100/100/NA
	Hits	99/72/68		98/72/74	98/95/NA
	Out	230/86/76		123/90/75	116/112/NA
	Matches	98/68/64		97/70/73	79/76/NA
KEGG	In	97/97/97	97/97/97		97/97/NA
	Hits	96/81/71	97/71/75		95/94/NA
	Out	224/101/80	114/71/75		111/116/NA
	Matches	95/79/69	97/70/73		77/86/NA
PubChemCID	In	100/100/NA	100/100/NA	100/100/NA	
	Hits	97/88/NA	98/76/NA	96/87/NA	
	Out	228/103/NA	117/76/NA	121/108/NA	
	Matches	96/88/NA	98/76/NA	95/87/NA	

Table S2: Results of each iteration of the additive identifier mapping tests. Results are given for CTS/UniChem. NA implies that the input identifier type, output identifier type, or both were not covered by the corresponding application. PubChem refers to the PubChem Compound database and KEGG to the KEGG Compound database.

Input type	Count	Output type: ChEBI	HMDB	KEGG	PubChem
Name only	In	100/NA	100/NA	100/NA	100/NA
	Hits	66/NA	67/NA	65/NA	70/NA
	Out	144/NA	78/NA	106/NA	235/NA
	Matches	59/NA	65/NA	63/NA	65/NA
+ InChIKey	In	100/100	100/100	100/100	100/NA
	Hits	90/68	98/100	92/73	100/NA
	Out	109/76	99/100	116/74	123/NA
	Matches	82/63	96/98	90/72	80/NA
+ ChEBI	In	100/100	100/100	100/100	100/NA
	Hits	90/68	98/100	96/84	100/NA
	Out	109/76	99/102	121/86	144/NA
	Matches	82/63	96/99	96/84	97/NA
+ HMDB	In	100/100	100/100	100/100	100/NA
	Hits	90/69	98/100	96/85	100/NA
	Out	108/77	99/102	119/86	125/NA
	Matches	81/64	96/99	95/84	82/NA
+ KEGG	In	100/100	100/100	100/100	100/NA
	Hits	93/77	98/100	96/85	100/NA
	Out	110/85	99/102	119/86	135/NA
	Matches	87/72	96/99	95/84	94/NA
+ PubChem	In	100/NA	100/NA	100/NA	100/NA
	Hits	94/NA	98/NA	96/NA	100/NA
	Out	112/NA	99/NA	121/NA	135/NA
	Matches	91/NA	96/NA	96/NA	94/NA

2 Step-by-step description of the annotation of Recon 2 metabolites

We used the CTS web service for mapping between identifiers (see http://cts.fiehnlab.ucdavis.edu/moreServices/index). We called the web service from MATLAB (version R2009b, MathWorks, Natick, MA), using the function urlread. The web service returns a JSON string, which we parsed for output identifiers using regular expressions (function regexp) in MATLAB.

1. Review existing identifiers in Recon 2

   i. Download metabolite structures from online databases for all identifiers in Recon 2.

   ii. Extract metabolite formulas from downloaded structures. We did this using version 5.12.3 of ChemAxon's Calculator Plugins (ChemAxon Kft., Budapest, Hungary).

   iii. If the metabolite formula extracted from a structure does not match the one in Recon 2, discard the corresponding identifier. Ignore differences in numbers of hydrogen atoms.

2. Map Recon 2 identifiers to HMDB ID with CTS

   i. Generate a list of all existing HMDB ID in Recon 2. This is the initial list of candidate HMDB ID for metabolites in Recon 2. Assign a confidence score of 0 to each HMDB ID in the list.

   ii. Map the first metabolite name in Recon 2 to HMDB ID. If one of the returned HMDB ID was already listed in the previous step as a candidate HMDB ID for the corresponding metabolite, add 0.5 to its confidence score. If new HMDB ID are returned, add them to the list of candidate HMDB ID and assign them a confidence score of 0.5. Repeat for all metabolite names in Recon 2.

   iii. Map the first standard InChIKey in Recon 2 to HMDB ID. If any of the returned HMDB ID were already listed in previous steps as candidate HMDB ID for the corresponding metabolite, add 1 to their

confidence score. If new HMDB ID are returned, add them to the list and assign them a confidence score of 1. Repeat for all standard InChIKeys in Recon 2.

iv. Map all ChEBI ID in Recon 2 to HMDB ID in the same way as standard InChIKeys were mapped.

v. Map all KEGG CID in Recon 2 to HMDB ID in the same way as standard InChIKeys were mapped.

vi. Map all PubChem CID in Recon 2 to HMDB ID in the same way as standard InChIKeys were mapped.

3. Rank candidate HMDB ID for each metabolite by their confidence scores.

4. Select preferred HMDB ID from the list of candidates

    i. For metabolites with an existing HMDB ID in Recon 2: If the existing HMDB ID is one of the top ranked HMDB ID for the corresponding metabolite, keep it automatically. If not, select preferred HMDB ID manually from the list of all candidate HMDB ID for the corresponding metabolite.

    ii. For remaining metabolites: If there is only one top ranked HMDB ID for a given metabolite, keep it automatically. Otherwise, select preferred HMDB ID manually from the list of top ranked HMDB ID for that metabolite.

5. Review new HMDB ID in Recon 2 by comparing formulas as in step 1.

6. Extract standard InChIKeys, ChEBI ID, KEGG CID and PubChem CID from HMDB. We did this by parsing MetaboCard Files (in XML format) for version 3.5 of HMDB. The MetaboCard files were downloaded from www.hmdb.ca/downloads.

7. Add identifiers extracted from HMDB to Recon 2 where necessary. Do not overwrite existing identifiers.

8. Review new ChEBI ID, KEGG CID and PubChem CID by comparing formulas as in step 1.

9. Map Recon 2 identifiers (old and new) to ChEBI ID, KEGG CID and PubChem CID with CTS. Follow the same procedure as for HMDB ID (step 2) for each type of output identifier, except using HMDB ID as input in place of the output type.

10. Rank candidate output identifiers by their confidence scores.

11. Select preferred ChEBI ID, KEGG CID and PubChem CID from the lists of candidates generated in steps 9 and 10, following the same procedure as for HMDB ID (step 4).

12. Review new identifiers in Recon 2 by comparing formulas as in step 1.

13. Map updated Recon 2 identifiers to LMSD ID following the same procedure as for HMDB ID (step 2) except with HMDB ID as an added type of input identifier.

14. Rank candidate output identifiers by their confidence scores.

15. Select preferred LMSD ID from the lists of candidates generated in steps 13 and 14, following the same procedure as for HMDB ID (step 4).

16. Review LMSD ID added to Recon 2 by comparing formulas as in step 1.
